# Access to quality health resources and environmental toxins affect the relationship between brain structure and BMI in a sample of pre and early adolescents

**DOI:** 10.3389/fpubh.2022.1061049

**Published:** 2022-12-15

**Authors:** Shana Adise, Andrew T. Marshall, Eric Kan, Elizabeth R. Sowell

**Affiliations:** ^1^Division of Pediatric Research Administration, Department of Pediatrics, Children's Hospital Los Angeles, Los Angeles, CA, United States; ^2^Division of Neurology, Department of Pediatrics, Children's Hospital Los Angeles, Los Angeles, CA, United States

**Keywords:** structural MRI, neighborhood deprivation, adolescence, built environment, area deprivation, pediatric obesity, health policy, structural brain development

## Abstract

**Background:**

Environmental resources are related to childhood obesity risk and altered brain development, but whether these relationships are stable or if they have sustained impact is unknown. Here, we utilized a multidimensional index of childhood neighborhood conditions to compare the influence of various social and environmental disparities (SED) on body mass index (BMI)-brain relationships over a 2-year period in early adolescence.

**Methods:**

Data were gathered the Adolescent Brain Cognitive Development Study^®^ (*n* = 2,970, 49.8% female, 69.1% White, no siblings). Structure magnetic resonance imaging (sMRI), anthropometrics, and demographic information were collected at baseline (9/10-years-old) and the 2-year-follow-up (11/12-years-old). Region of interest (ROIs; 68 cortical, 18 subcortical) estimates of cortical thickness and subcortical volume were extracted from sMRI T_1_w images using the Desikan atlas. Residential addresses at baseline were used to obtain geocoded estimates of SEDs from 3 domains of childhood opportunity index (COI): healthy environment (COI_HE_), social/economic (COI_SE_), and education (COI_ED_). Nested, random-effects mixed models were conducted to evaluate relationships of BMI with (1) ROI ^*^ COI_[domain]_ and (2) ROI ^*^ COI_[domain]_^*^ Time. Models controlled for sex, race, ethnicity, puberty, and the other two COI domains of non-interest, allowing us to estimate the unique variance explained by each domain and its interaction with ROI and time.

**Results:**

Youth living in areas with lower COI_SE_ and COI_ED_ scores were heavier at the 2-year follow-up than baseline and exhibited greater thinning in the bilateral occipital cortex between visits. Lower COI_SE_ scores corresponded with larger volume of the bilateral caudate and greater BMI at the 2-year follow-up. COI_HE_ scores showed the greatest associations (*n* = 20 ROIs) with brain-BMI relationships: youth living in areas with lower COI_HE_ had thinner cortices in prefrontal regions and larger volumes of the left pallidum and Ventral DC. Time did not moderate the COI_HE_ x ROI interaction for any brain region during the examined 2-year period. Findings were independent of family income (i.e., income-to-needs).

**Conclusion:**

Collectively our findings demonstrate that neighborhood SEDs for health-promoting resources play a particularly important role in moderating relationships between brain and BMI in early adolescence regardless of family-level financial resources.

## Introduction

It is becoming increasingly clear that the social and built environments in which children live, play, and study is an important predictor of health outcomes and cognitive functioning. This combination of social and environmental resources refers to the social determinants of health (SDOH) (1). SDOH influence health from several levels: (a) macrosocial (e.g., sociopolitical, sociocultural); (b) neighborhood resources (e.g., food environment [e.g., distribution, food deserts, food insecurity, price]); (c) natural and built environment infrastructure (e.g., urban design, transportation, land use); (d) social environment (e.g., poverty, living conditions, remoteness, eating culture); and (e) individual social/economic level (e.g., income, education, race/ethnicity). Combined, each of these components are proposed to drive greater intake of unhealthy foods and obesity within communities. Studies have shown that several of these determinants play a role in optimal child development starting from conception, as the environment and its resources have been linked to early life outcomes, such as low birthweight, preterm delivery, and gestational problems (2, 3). However, SDOH continue to affect health throughout the lifespan, with studies showing associations with other health outcomes, such as childhood obesity (4), cardiovascular disease risk (5), and brain structure and cognition (6, 7). Childhood obesity rates have continued to increase (8), despite prevention and intervention efforts, suggesting that the mechanisms driving this disease are poorly understood. Because the risk of obesity starts *in utero* (9), and the influence of SDOH on health outcomes starts at conception (2, 3), there is dire need to understand how social and environmental resources impact risk and obesity development.

Traditionally, SDOH have been studied by assessing how one single attribute in the environment relates to obesity risk. For example, poverty [e.g., low income (10)], parental education (11), access to grocery stores, and green spaces (12) have separately been related to childhood obesity. Although informative, such isolated analyses *via* single attributes do not capture how other conditions may mitigate risks associated with living in disadvantaged neighborhoods. For example, some social and environmental factors may serve as protective factors (e.g., access to education, healthy food choices) and mitigate the risks of detrimental factors (e.g., poverty). As diseases, like childhood obesity, are multifactorial (13), there is need to apply a multifactorial environmental approach to understand how the environment collectively across numerous domains influences obesity in consideration of SDOH. Additionally, many of these single attributes are confounded by racial and ethnic groups experiencing disparities. Racial and ethnic minorities are more likely to live in neighborhoods with fewer resources (14), more likely to earn lower incomes (15), less likely to obtain higher education (16), and have higher rates of obesity and other health comorbidities (17). Given these biases, it is unclear if single-attribute data are sufficient markers for SDOH.

Multiple SDOH attributes can be assessed by multidimensional composite scores that are designed to study social and environmental disparities (SED), such as the Child Opportunity Index (COI; https://www.diversitydatakids.org/child-opportunity-index) (18). SED are closely related to the SDOH, but focus on geocoded, multidimensional composite scores based on neighborhood-level estimates of environmental resources that are closely linked to optimal childhood health. Multidimensional composite scores offer greater explanatory power and are more accurate assessments of a community than single attribute measures (19). The COI is a multidimensional population-level surveillance tool, and the data are publicly available. It incorporates multiple neighborhood-level factors that are specific to healthy child development, such as neighborhood poverty, air pollution, education access, healthy food store access, and the number of surrounding green spaces. These neighborhood factors can be summed into an overall index or treated separately by domain (e.g., education, health/environmental, social/economic), ultimately permitting understanding as to how neighborhood conditions, which have been shown to affect child development, are associated with other aspects of healthy development.

The COI has been used across varying domains to establish links between SED and child health outcomes like cardiovascular disease risk (5), hospitalizations for acute ambulatory care (20), and pediatric acute care (21). Other broader indices, like the area deprivation index (ADI), have been used to assess how neighborhood deprivation relates to childhood obesity (6, 22, 23). However, broad indices like ADI do not provide insight into how necessary health resources relate to childhood obesity; for example, the ADI does not assess other neighborhood factors, such as grocery store access, air pollution, or educational resources available to children. Moreover, the ADI was not created based on neighborhood data that were specific to child development (e.g., school poverty, early childhood education). Thus, the goal of this study was to examine how different aspects of social and environmental resources that are specifically related to childhood development (e.g., COI; domains: education, health/environmental, social/economic resources) are associated with childhood obesity.

Using the COI, we aimed to determine the variance explained by one domain (e.g., health/environmental) and its interaction with other factors while accounting for the variance explained by the other two domains (e.g., education, social/economic). In doing so, the goal of this study was to understand the importance of each domain, independently from the other domains for childhood obesity. For several reasons, the analyses focused on how SED moderate the relationship between body mass index (BMI) and structural brain development during adolescence. First, altered brain structure has been observed in youth with obesity (24), suggesting that one reason for overeating is impaired neural signaling around food choice. Because the brain plays a key role in food-intake regulation (25), it is imperative to understand more about the brain-environment relationships that may continue to escalate obesity risks. Second, SED also impact brain development, and inadequate resources have been correlated with altered brain structure as well (7, 26, 27). However, it is not known how each of these domains may further interact with the brain to influence obesity risk. Moreover, to our knowledge, no studies have looked at how multiple environmental factors relate to obesity risk and brain structure development over time. Therefore, it is not known whether these associations are stable (e.g., social and environmental aspects have a general effect that does not worsen over time) or if they have a sustained impact throughout development. To address these questions, we utilized data from the baseline and 2-year follow-up from the Adolescent Brain Cognitive Development study (ABCD Study^®^), a 10-year longitudinal neuroimaging study in 11,878 American youth (28).

## Methods

### Study design

The ABCD Study^®^ is 10-year, multiple site longitudinal cohort study being conducted in 11,878 American youth; several publications have described the goals, design, and assessments (29–35). The overall goal of this observational cohort study was to assess development across a range of metrics (e.g., brain development, cognition, substance abuse, mental health). A list of assessments and their collection year can be viewed at (www.ABCDStudy.org). Recruitment for the study aimed to reflect the demographic estimates of the United States; participants were recruited through schools. Recruitment and design considerations to maximize data collection across various populations are detailed elsewhere (29). At the start of the ABCD Study^®^, youth were 9–10-years-old, and assessments were conducted yearly thereafter. The current manuscript presents data from the 4.0 release and includes anthropometric and neuroimaging assessments at baseline (ages 9–10-years-old) and the 2-year follow-up (ages 11–12-years-old). The COI index was generated using residential history provided by the caregiver at the baseline assessment. Caregivers and youth provided written consent and assent. A centralized institutional review was approved by the University of San Diego.

### Exclusion criteria

The larger ABCD Study^®^ eligibility was generally inclusive, but some exclusions were applied such as: MRI contraindications (e.g., non-removable metal implants, dental appliances), not fluent in English (child only), a history of major neurological disorders (e.g., current diagnosis of schizophrenia, mental retardations, autism spectrum disorder (moderate/severe), pre-maturity at birth <28 weeks and/or hospitalization at birth >30 days, uncorrected vision, known alcohol or substance abuse problems. Additional exclusions were applied to obtain an optimal sample for the present analyses and hypotheses. Specifically, across any time point (baseline, two-year follow-up), the exclusion criteria consisted of: (a) Underweight (according to the Center for Disease Control's (CDC's) age-sex-height-weight-specific growth curves) (36) as youth who are in this category were removed to avoid inclusion of those with potential restrictive eating or medical issues to make them underweight; (b) medications known to alter food intake (e.g., antipsychotics, insulin); (c) caregiver report of neurological, psychiatric, or learning disabilities; (d) they met diagnostic criteria for eating disorders (e.g., anorexia, binge eating disorder) as assessed by the caregiver-reported Kiddie Schedule for Affective Disorders and Schizophrenia (37); (e) mislabeled sex-assigned at birth and/or mismatched sex-specific pubertal questionnaires or transgender youth so that there were no inconsistencies in any sex-specific effects on brain structure; (f) height measurement error (e.g., height at year 2 less than height at baseline); (g) missing income data; (h) invalid residential address (necessary for geocoded metrics); (i) failed FreeSurfer segmentation; (j) failed T_1_-weighted image quality control; and/or (k) missing ROI or covariate tabulated data from the National Institutes of Mental Health Data Archives. Siblings were excluded to avoid issues with independence. The final sample consisted of 2,749 youth (Table 1).

**Table 1:** Participant characteristics.

	**Subsample**		**Entire ABCD study**^**^®^**^ **cohort**		
**Variable**	**Mean**	**SD**	**Mean**	**SD**	***p***
**Age**					
Baseline	118.9	7.4	119	7.5	0.699
Y2	142.9	7.6	143.8	7.9	<0.001
**Puberty**					
Baseline	2	0.8	2	0.8	0.372
Y2	2.7	1.0	2.7	1	0.067
**BMI**					
Baseline	19	3.7	19.4	41.7	0.421
Y2	21	4.6	21.5	50.3	0.533
Income to needs ratio	3.9	2.4	3.6	2.4	<0.001
	***n***	**%**	***n***	**%**	
**Sex**					
Male	1,381	50.2	4,812	52.7	0.047
Female	1,368	49.8	4,316	47.3	
**Race**					
White	1,944	70.7	5,581	62.3	<0.001
Black	298	10.8	1,572	17.5	
Asian	64	2.3	211	2.4	
AIAN/NHPI	25	0.9	53	0.6	
Other	116	4.2	409	4.6	
Multi-race	302	11	1,132	12.6	
**Ethnicity**					
Hispanic	525	19.1	1,886	21	0.032
Non-Hispanic	2,224	80.9	7,090	79	
**Caregiver report of education**					
<HS	74	2.7	295	3.2	<0.001
HS/GED	177	6.4	494	5.4	
Some college	644	23.4	903	9.9	
BA degree	749	27.2	2,334	25.6	
Postgraduate degree	1,105	40.2	2,220	24.3	
**Baseline weight class**					
Healthy weight	1,849	67.3	5,753	63	<0.001
Overweight	462	16.8	1,340	14.7	
Obese	438	15.9	1,554	17	
**Y2 weight class**					
Healthy weight	1,716	64.1	3,058	39.9	<0.001
Overweight	482	17.5	735	9.6	
Obese	506	18.4	875	11.4	

Baseline was assessed when the youth were 9–10-years-old. Y2, two-year follow up; BMI, Body mass index; AIAN/NHPI, American Indian, Alaskan Native/Native Hawaiian, and Pacific Islanders; HS, high school; GED, Generalized education diploma; BA, Bachelor's degree; SD, standard deviation. P-values were generated from t-tests and chi-square analyses were appropriate. Education data were gathered from the baseline assessment.

### Anthropometrics

A trained researcher measured the youth's height (nearest 0.1 in/0.25 cm) and weight (nearest 0.1 lb/0.045 kg) twice, but a third measurement was collected in cases of discrepancy. The closest two measurements were averaged and converted into BMI (kg/m^2^) and BMI percentiles according to the CDC's sex-age-height-weight specific growth charts (36). Given the biases surrounding z-score and percentiles (38, 39), these are provided only for clinical interpretations while BMI was used in the statistical analyses.

### Pubertal assessment

Both the youth and caregiver completed sex-specific puberty questionnaires. Responses were converted into Tanner stages (1 = Pre-pubertal, 2 = Early puberty; 3 = Mid puberty; 4 = Late puberty; 5 = Post-pubertal), and caregiver and youth reports were averaged.

### Demographic assessments

Race, ethnicity, date of birth, education, household income and sex at birth were reported by the attending caregiver. Caregiver reported race had 22 options, which were collapsed into six groups: White; Black; Asian; American Indian, Alaskan Native/Native Hawaiian, Pacific Islander; Other; multi-race. Caregiver reported ethnicity was assessed with two options: Hispanic or Non-Hispanic. Age at each visit was recorded in months. There were 29 responses for household education levels, which were collapsed into five groups: <High school (HS; <13 years), HS/Generalized Education Diploma (~13 years), Some college (<2 years post HS), 4 year degree (Bachelor's degree post HS), Postgraduate education (>4 years post HS). There were 10 responses for household income: (a) <$5,000; (b) $5,000 – $11,999; (c) $12,000-$15,999; (d) $16,000-$24,999; (e) $25,00-$34,999; (f) $35,000-$49,999; (g) $50,000-$74,999; (h) $75,000-$99,999; (i) $100,000–$199,999; (j) >$20,000. There were also options for (a) don't know or (b) refuse to answer. However, data with responses that included “I don't know” or “Refuse to answer” were omitted.

### Income-to-needs

Income-to-needs was calculated as the ratio between the total household income and the poverty rate per household size. Poverty rates were calculated according to the 2017 Department of Health and Human Services' report of poverty level (40). The 2017 report was used because baseline assessments for the ABCD Study^®^ were conducted between 2016 and 2018.

### Child opportunity index

The COI is an index of neighborhood resource availability that is beneficial for healthy child development (18). An overall composite score is computed based on 29 neighborhood indicators that have clinical relevance. Each indicator had an individual weight that was based on how strongly the indicator predict health and economic outcomes. Domain scores were combined using individually weighted indicators where the weights reflected the strength of the association between each indicator and its health and socioeconomic outcome. COI composite scores were calculated by the data analytics and informatics resource core (DAIRC) of the ABCD Study^®^ in R (https://github.com/ABCD-STUDY/geocoding/blob/master/Gen_data_proc.R) (41).

The geocoded data used to create the COI provides information for almost all United States neighborhoods (~72,000 census tracts from 2010 and 2015). For each census track neighborhood, a composite score is computed separately for three domains: education (COI_ED_), health/environmental (COI_HE_), and social/economic (COI_SE_). The COI_ED_ domain reflects the quality and access to early childhood education, quality of schools, and resources related to academic achievement. The COI_HE_ domain reflect the number of features of the healthy environment, such as access to green spaces and healthy foods, as well as environmental toxicant exposure. The COI_SE_ domain comprises metrics geared at assessing access to employment as well as neighborhood social/economic resources. Scores on each of these domains range from 1 (lowest) to 100 (highest); lower composite scores indicate fewer available resources. Domain composite scores were transformed to a *z*-score to compare neighborhoods and features over time. In addition, these *z*-scores were adjusted to reflect national norms (18). The nationally normed, raw score across all domains [range 1–100 units (U)] was transformed into 5 categories to contextualize the relative level of opportunities in the neighborhood: very low (<20 U), low (≥20– <40 U), moderate (≥40– <60 U), high (≥60– <80 U), and very high (≥80 U) opportunities (14). In this instance, national norms are constructed by raking all neighborhoods nationwide and diving them into five groups, each containing 20% of the child population.

### Neuroimaging acquisition and processing

The ABCD Study^®^ collected T_1_- and T_2_-weighted MRI, diffusion tensor imaging, resting state MRI, and three functional MRI scans at baseline and the 2-year follow-up. The current manuscript only includes the T_1_-weighted structural, imaging acquisition. The ABCD Study^®^ DAIRC was responsible for MRI data preprocessing and analyses. These methods are published elsewhere (35, 42) but described in brief here: After preprocessing, cortical data were surface projected and then parcellated with Freesurfer using the Desikan Atlas (42), which consists of 68 regions of interest (ROIs). Subcortical data were parcellated from the volumetric data. ROI estimates (e.g., mean cortical thinning, total gray matter volumes) were made available through the tabulated data release. ROI estimates were averaged across hemispheres (e.g., left, right), for a total of 34 cortical and 8 subcortical ROI estimates. Across the 21-sites, data were collected using 29 scanners, as some sites had multiple MRI acquisition centers. The ABCD Study^®^ Release Notes provide recommended inclusion criteria calculated by the DAIRC for the user to apply (e.g., T1-weighted image passed quality control assessment: 0 = no, 1 = yes).

### Linear mixed-effects modeling

Data were preprocessed in Python and mixed effects were conduted using the pymer4 package (43). Prior to analyses, data went through minimal preprocessing: (a) the top and bottom 5% of data were winsorized to restrict outliers in the ROI-level data; (b) continuous data were standardized to compare variables with different scales and for interaction interpretation. Next, multicollinearity was assessed using a variance inflation factor. After these preprocessing steps, linear mixed effects analyses were conducted. The dependent variable was BMI. The independent variable consisted of the COI domain of interest: (a) COI_ED_, (b) COI_HE_, or (c) COI_SE_. Three-way (e.g., Brain ^*^ COI ^*^ Time) and two-way (e.g., Brain ^*^ COI) interactions were included for each of the three domains in separate models. The models were covaried for Youth sex, Youth's caregiver reported race, Youth's caregiver reported ethnicity, puberty, time (e.g., baseline, two-year follow-up visit), income-to-needs ratio, and the two other COI domains of *no interest*. For example, if the primary independent variable was the COI_ED_, main effects were also modeled for COI_HE_ and COI_SE_ (i.e., COI domains of no interest) to account for variance explained by these other domains. Covarying for the other COI domains allowed us to examine the unique variance of each domain while accounting for the others, but the aforementioned interactions in each model were included for that model's COI domain of interest.

Additionally, the model included a random intercept for MRI scanner serial number (*n* = 29, to account for the number of scanners across the 21 sites), and a random slope for subject. Factors were specified using effects coding. Of importance, our analyses controlled for family-specific income-to-needs ratio, which allowed for us to examine the relationship between neighborhood economics (COI) statistically independent from family economics (income-to-needs). The Benjamini-Hochberg approach (44) was used to correct for multiple comparisons for each brain measure separately (e.g., cortical thickness, subcortical volume). Correction was applied to only interaction terms of interest: (a) Brain ^*^ COI and (b) Brain ^*^ COI ^*^ Time. Thus, the correction for cortical data resulted in 136 corrections, while the subcortical data had 28 corrections.


$$\begin{array}{l} \text{MODEL}:\text{BMI }\sim \text{ Brain }*\;{\text{COI}}_{1}\text{ + Brain }*\;{\text{COI}}_{1}\;*\text{ Time}\\ \text{ + sex + puberty + race + ethnicity}\\ \text{ + }{\text{COI}}_{2}\text{ + }{\text{COI}}_{3}\text{ + }(\text{1| scanner/subject}). \end{array}$$

Further, our models included factor terms for youth's caregiver reported race and ethnicity because youth from these families have been shown to be more likely to live in deprived neighborhoods (14), in that COI, as a geocoded metric, may be skewed by historical marginalization and inequalities across neighborhoods; thus, inclusion of self-reported race and ethnicity is often included as factors in health disparity and geocoded analyses [see Acevedo-Garcia et al. (14), Noelke et al. (45), and Slopen et al. (46)]. Thus, we also chose to control for youth's caregiver-reported race and ethnicity in the models to accounts for historical issues surrounding race and ethnicity and resource deprivation as well as differences in disparities across race and ethnicity that exist in body composition (whether due to environmental, societal, and/or cultural factors differences) (47). However, because this paper was not specifically focused on evaluating potential differences across different races and ethnicities, we did not (and encourage readers to not) interpret such results. Further, in line with best practices (48), our model also included other measures that touch on SDOH and SED, such as income-to-needs and caregiver education. Accordingly, by including all these metrics in the model, we aimed to remove within-family variance that may influence our primary outcome (e.g., BMI). A larger and continued discussion of this is included in the limitations section at the end of the manuscript.

## Results

### Demographics

The sample included in the analyses consisted of 2,749 youth (Table 1). The mean age at baseline was 118.9 months ± 7.4 and 142.9 months ± 7.6 at the 2-year follow-up. The sample was 50% male (n=1,381), 70% caregiver reported White, 19.1% caregiver reported Hispanic, and 67.4% of youth had caregivers with an education of at least a Bachelor's Degree or higher. The parent-reported demographics of the youth included in the sample was similar to the larger dataset. Estimates of healthy weight, overweight and obesity were similar to national estimates in the USA (49).

### Distribution of the COI across participants

**Figure 1:**
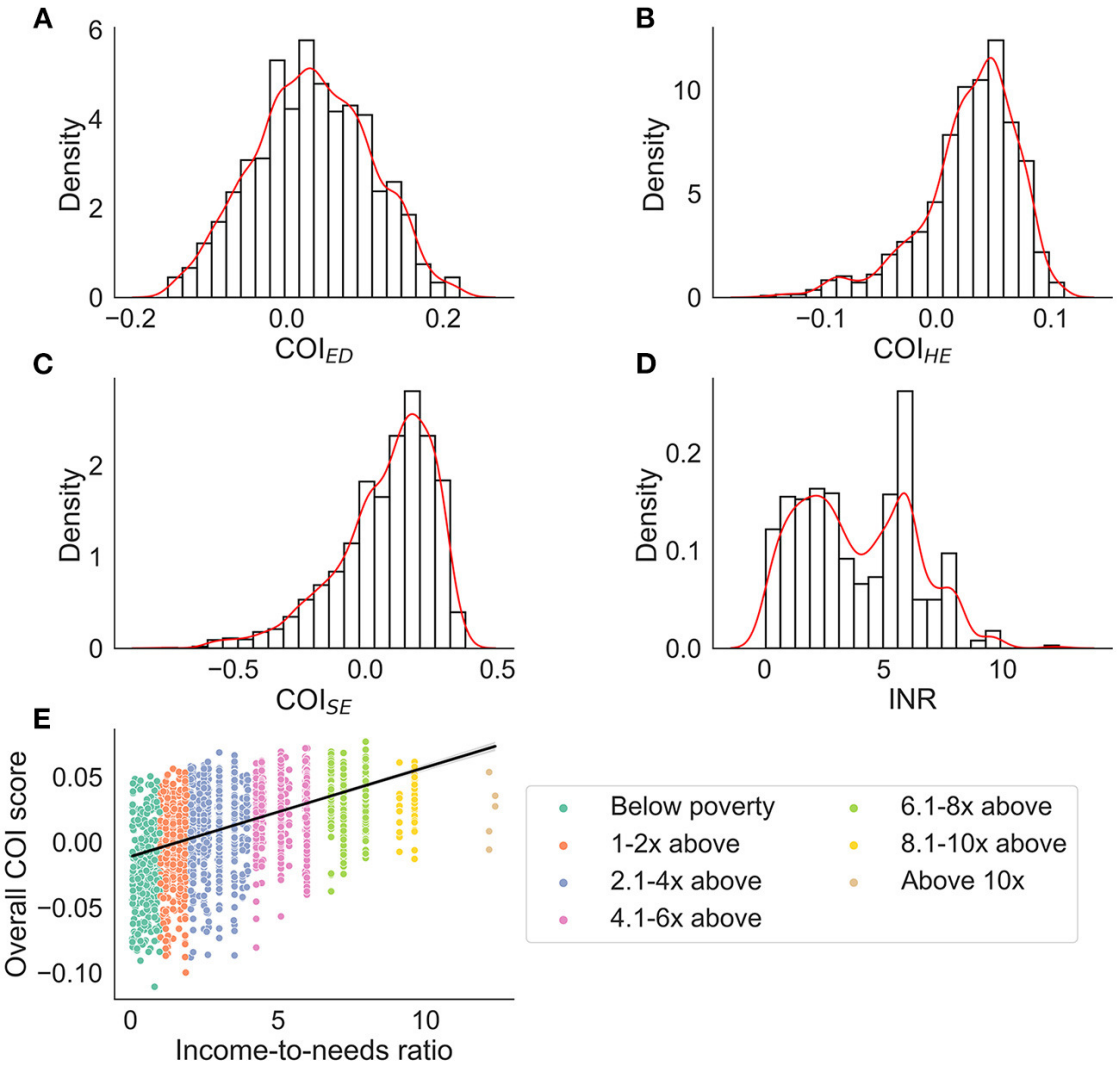
Distributions for each COI domain (nationally normed and z-score transformed) and income-to-needs ratio (INR). For **(A–D)** density (i.e., probability generated from the Kernel Density Estimation) is plotted on the y-axis. The red line represents a smoothed distribution. **(E)** The overall COI score binned by income-to-needs ratio to represent how many youth were below or above the poverty line. ED, education domain; HE, health/environmental domain; SE, social/economics domain.

The distribution of each COI domain and income-to-needs are presented in Figure 1. Across all COI domains and with respect to national norms, 10.8% of youth lived in very low opportunity neighborhoods (<20 U), 12.1% lived in low opportunity neighborhoods (≥20– <40 U), 14.8% lived in moderate opportunity neighborhoods (≥40– <60 U), 25.0% lived in high opportunity neighborhoods (≥60– <80), and 37.3% lived in very high opportunity neighborhoods (≥80 U). Youth included in the analyzes had slightly higher opportunities across all domains than the larger ABCD Study^®^ sample (Table 2). Although we chose not to focus on self-reported racial and ethnic disparities and resource access, we have provided a visual representation of how these metrics relate to resources within the neighborhood by race (Supplementary Figure 1), which shows the distribution of resources across each domain by self-reported race and ethnicity; although youth from self-reported racial minorities were more likely to live in neighborhoods with fewer resources, resource disparity is observed across all self-reported races and ethnicities. Moreover, the ABCD Study^®^ sample was large enough to be able to adequately account for these differences. Thus, Figure 1 is purely displayed for the reader to understand how disparities affect communities rather than for interpretation that can lead to racist misinformation and misinterpretation.

**Table 2:** Child opportunity index (COI) scores.

	**Sample included in the analyses**		**Entire ABCD Study**^**^®^**^ **cohort**		
**COI**	**Mean**	**SD**	**Mean**	**SD**	***p***
COI_ED_	63.1	28.3	59.3	30.2	<0.001
COI_HE_	60.9	29.2	57.6	30.4	<0.001
COI_SE_	62.5	28.9	58.1	31.3	<0.001
Overall COI opportunity	63.4	28.8	58.9	31.3	<0.001
	**n**	**%**	**n**	**%**	
Overall COI opportunity level					
Very low	352	12.8	1,466	18.2	<0.001
Low	294	10.7	998	12.4	
Moderate	417	15.2	1,109	13.8	
High	797	25.7	1,827	22.7	
Very high	979	35.6	2,636	32.8	

Mean and standard deviation (SD) are reported for each Child Opportunity Index (COI) domain. Overall opportunity by level is provided for a contextual reference and interpretation. P-values correspond to t-tests and chi-square analyses where appropriate. ED, education domain; HE, health/environmental domain; SE, social/economics domain.

### Brain ^*^ COI^*^ Time effects

#### COI_ED_

**Figure 2:**
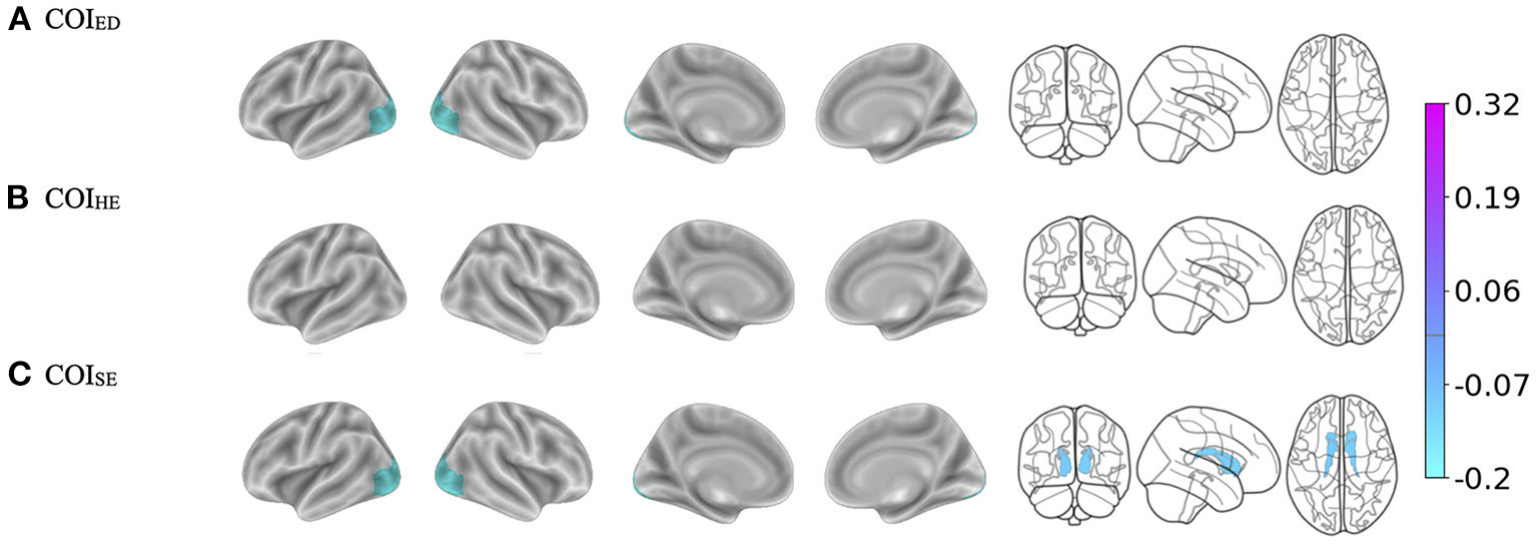
**(A–C)** Visualization of the brain regions that showed significant three-way interactions with each Child Opportunity Index (COI) domain of interest by time (e.g., baseline, 2-year follow-up), controlling for the other two COI domains of no interest, income-to-needs, sex, puberty, race, and ethnicity. The color bar corresponds to the regression coefficients from the mixed models. There are no effects in **(B)** but the brain template is reported for ease of comparison. ED, education domain; HE, health/environmental domain; SE, social/economics domain.

**Figure 3:**
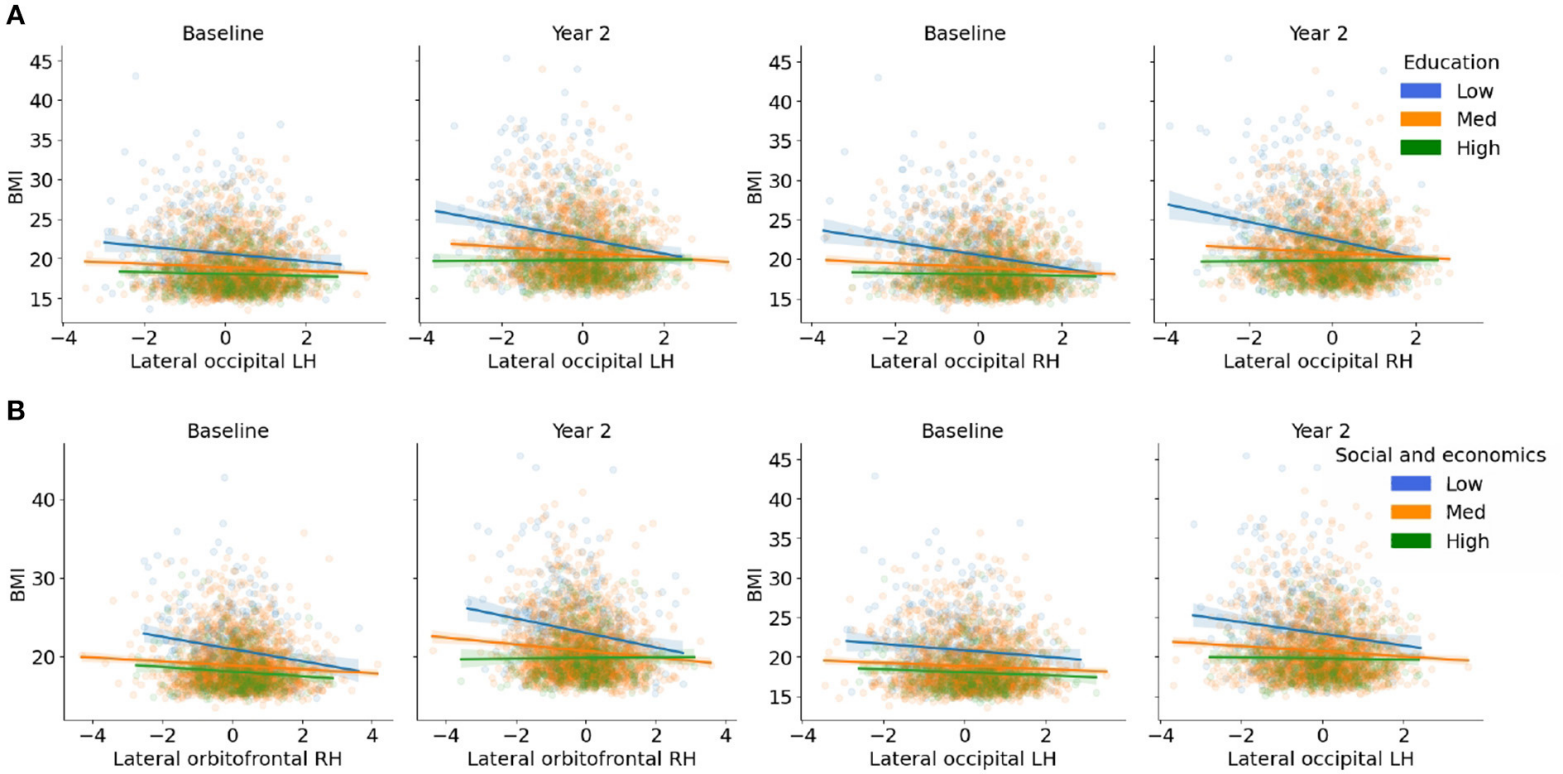
**(A,B)** Visualization of the three-way interactions between the brain, each Child Opportunity Index (COI) domain of interest, and time (e.g., visit number [baseline, year-two follow-up]), controlling for the other two COI domains of no interest, income-to-needs, sex, puberty, race, and ethnicity. The top two Brain * COI * Time interactions are plotted for each domain. The y-axis presents the body mass index (BMI) predicted fits from the regression. **(A)** COI_ED_; **(B)** COI_SE_. Low (blue), medium (orange), and high (green) correspond to the values of each COI at −1 standard deviation (SD) below the mean, at the mean, and 1 SD above the mean. Fit lines indicate the regression fit with a 95% confidence interval. Individual points (i.e., subjects) are represented by the dots for each color. Lower values of each COI indicate that there were fewer resources in the area. RH, right hemisphere; LH, left hemisphere.

There were three-way interactions on BMI between the brain, COI_ED_, and time in the bilateral lateral occipital cortices [Left: *F*_(1, 1, 039.1)_ = 15.1, *p* < 0.001; Right: (*F*_(1, 1, 063.4)_ = 14.9, *p* < 0.001), Figure 2A, Table 3]. Although the overall pattern suggests that youth with access to fewer quality educational resources have greater BMI's and thinner cortices, this relationship was stronger at the 2-year follow-up. That is, at the 2-year follow-up, youth with access to fewer quality educational resources showed even greater negative associations between increased BMI and decreased cortical thickness in the bilateral occipital cortex. Visual representations for the interactions for the two largest effects are presented in Figure 3A. These interaction effects were independent of Youth sex, Youth ethnicity, Youth race, puberty, income-to-needs, time (e.g., baseline, 2-year follow-up), COI_HE_ and COI_SE_ resources of the neighborhood.

**Table 3:** Brain regions that showed three-way interactions with each domain of the child opportunity index (COI).

**ROI**	***F***	***p***	**Beta**	**95% CI**
**COI**_**ED**_				
**Cortical thickness**				
Lateral occipital LH	14.87	0.0***^a^	−0.261	[−0.394, −0.128]
Lateral occipital RH	15.11	0.0***^a^	−0.263	[−0.396, −0.131]
**COI**_**SE**_				
**Cortical thickness**				
Lateral occipital LH	15.48	0.0***^a^	−0.266	[−0.399, −0.134]
Lateral occipital RH	17.21	0.0***^a^	−0.275	[−0.405, −0.145]
**Subcortical volume**				
Caudate LH	9.27	0.002**^a^	−0.188	[−0.309, −0.067]
Caudate RH	10.84	0.001***^a^	−0.202	[−0.323, −0.082]

Regions of interest (ROIs) were corrected separately for each modality (e.g., cortical thickness, subcortical volume) and accounted for the number of tests conducted (e.g., two-way and three-way interactions). Total corrections for cortical ROIs = 136 Total corrections for subcortical ROIs = 32. LH, left hemisphere; RH, right hemisphere. ^***^p <0.001; ^**^p <0.01; ^a^Benjamini-Hochberg FDR corrected. ED, education domain; HE, health/environmental domain; SE, social/economics domain.

#### COI_HE_

There were no three-way interactions observed (Brain ^*^ COI_HE_^*^ Time on BMI) (Figure 2B).

#### COI_SE_

There were three-way interactions (i.e., Brain ^*^ COI_SE_^*^ Time) on BMI for cortical thinning of the bilateral lateral occipital cortex [Left: *F*_(1, 964.6)_ = 17.2, *p* < 0.001; Right: (*F*_(1, 1, 048.8)_ = 15.5, *p* < 0.001)] and subcortical volume of the bilateral caudate [Left: *F*_(1, 900.7)_ = 10.5, *p* = 0.001; Right: (*F*_(1, 895.6)_ = 9.2, *p* = 0.002), Figure 2C, Table 3]. When compared to baseline, youth with access to fewer COI_SE_ resources showed steeper negative relationships between greater BMI and cortical thinning by the 2-year follow-up. Visualizations for the two most statistically significant three-way interactions are displayed in Figure 3B. These interaction effects were independent of Youth sex, Youth race, Youth ethnicity, puberty, income-to-needs, time (e.g., baseline, 2-year follow-up), and COI_HE_ and COI_ED_ resources of the neighborhood.

### Brain ^*^ COI effects

#### COI_ED_

**Figure 4:**
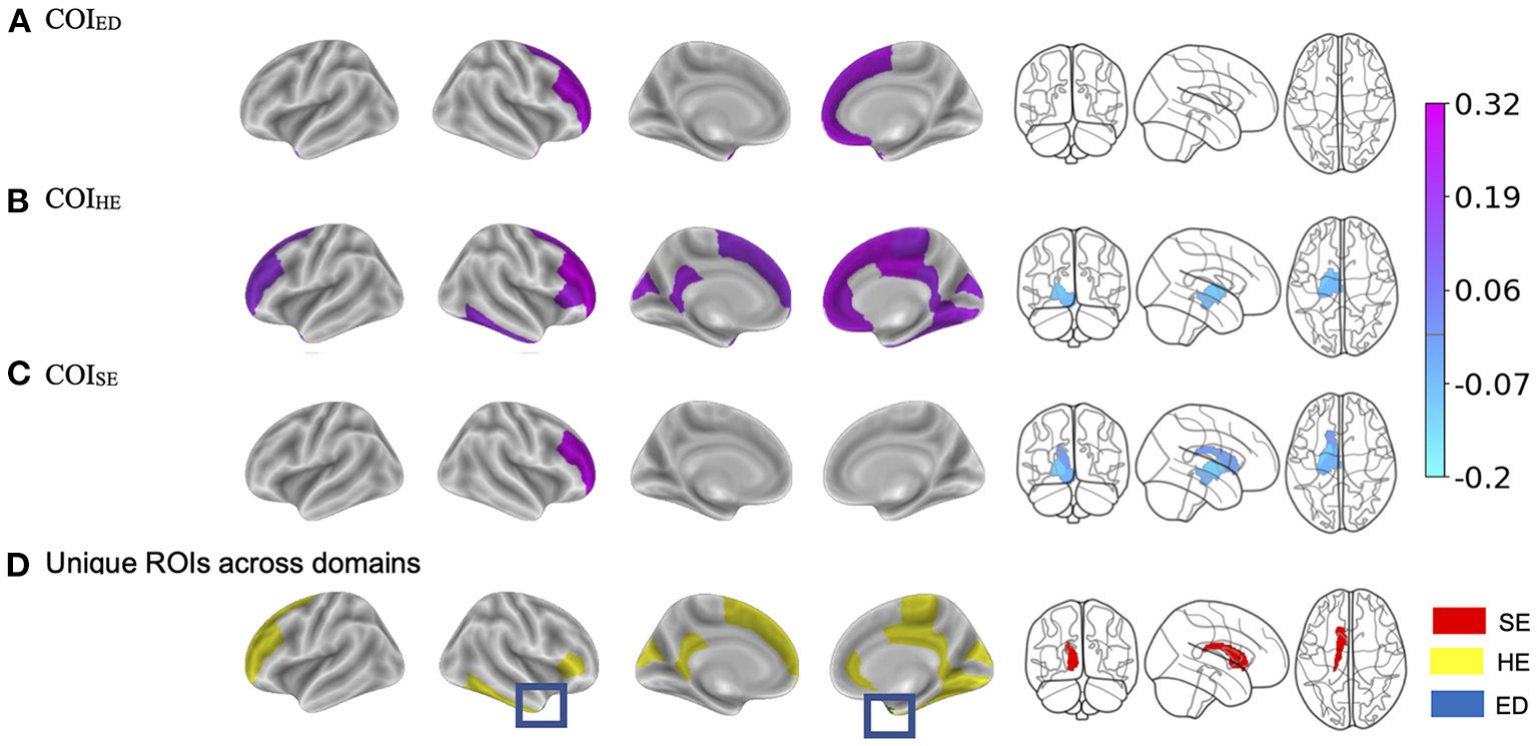
**(A–C)** Visualization of the brain regions that showed significant two-way interactions with each Child Opportunity Index (COI) domain of interest, controlling for the other two COI domains of no interest, income-to-needs, sex, puberty, race, ethnicity, and time (e.g., visit number [baseline, year-two follow-up]). The color bar corresponds to the beta weight from the mixed models. **(D)** Highlights the brain regions that showed unique associations per COI model that were not associated with another COI domain. SE, social/economic; HE, health/environmental; ED, education. The blue box highlights the region that was unique to the education domain. The color bar for **(A–C)**, corresponds to the regression coefficients.

**Figure 5:**
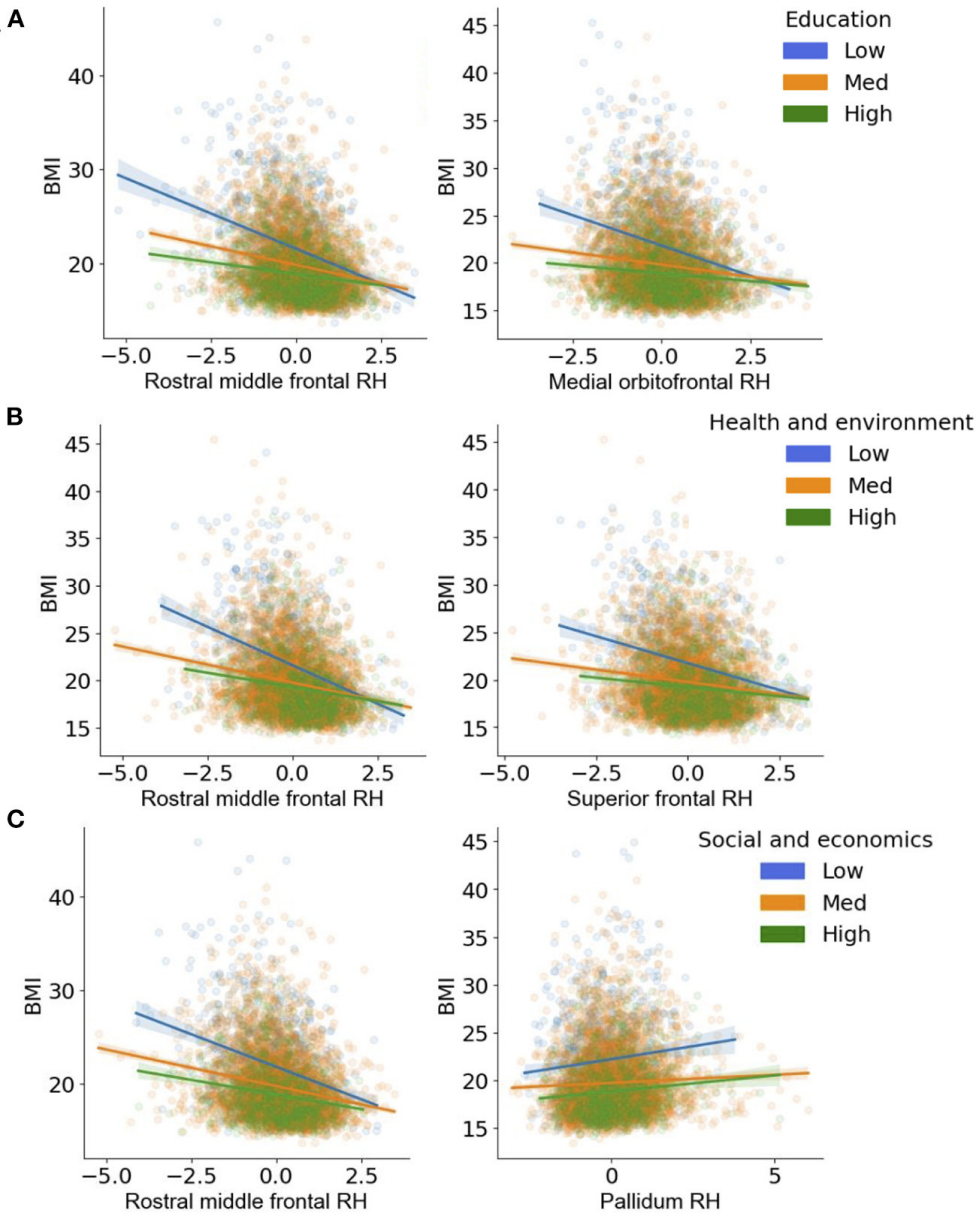
**(A–C)** Visualization of the two-way interactions between the brain and each Child Opportunity Index (COI) domain of interest, while controlling for the other two COI domains of no interest, as well as income-to-needs, sex, puberty, race, ethnicity, and time (e.g., visit number [baseline, year-two follow-up]). The top two Brain * COI interactions are plotted for each domain. The y-axis presents the body mass index (BMI) predicted fits from the regression. **(A)** education [ED]; **(B)** health/environmental [HE]; **(C)** social/economics [SE]. Low (blue), medium (orange), and high (green) correspond to the values of each COI at −1 standard deviation (SD) below the mean, at the mean, and 1 SD above the mean. Fit lines indicate the regression fit with a 95% confidence interval. Individual dots represent individual subjects collapsed over time. Lower values of each COI indicate that there were fewer resources in the area. RH, right.

Regardless of time, a child's access to quality education in the neighborhood (e.g., COI_ED_, access to early childhood education, quality of schools, resources allotted to academic achievement) moderated the strength of the relationship between BMI and cortical thickness in five regions (Figure 4A, Table 4). Specifically, children with access to fewer quality education resources in their neighborhood (lower COI_ED_) and who had greater BMI also had thinner cortices of the right medial orbitofrontal cortex [*F*_(1, 2, 538.1)_ = 15.6, *p* < 0.001], middle frontal gyrus [*F*_(1, 2, 766.5)_ = 14.5, *p* < 0.001], superior frontal gyrus [*F*_(1, 3, 406.2)_ = 12.6, *p* < 0.001], and the bilateral temporal pole [Left: *F*_(1, 2, 370.2)_ = 11.4, *p* < 0.001; Right: (*F*_(1, 2, 084.6)_ = 11.4, *p* < 0.001)]. Visual representations for the interactions for the two largest moderation effects are presented in Figure 5A. These moderation effects (e.g., ${\text{COI}}_{\text{ED}}^{*}$Brain on BMI) were independent of Youth sex, Youth race, Youth ethnicity, puberty, income-to-needs, time (e.g., baseline, 2-year follow-up), and the child's access to COI_HE_ and COI_SE_ resources of the neighborhood.

**Table 4:** Brain regions that showed two-way interactions with each domain of the child opportunity index (COI).

**ROI**	***F***	***p***	**Beta**	**95% CI**
**COI**_**ED**_				
**Cortical thickness**				
Medial orbitofrontal RH	15.63	0.000***^a^	0.262	[0.168, 0.356]
Rostral middle frontal RH	14.46	0.000***^a^	0.284	[0.192, 0.375]
Superior frontal RH	12.65	0.000***^a^	0.24	[0.145, 0.335]
Temporal pole LH	11.33	0.001***^a^	0.239	[0.146, 0.332]
Temporal pole RH	11.41	0.001***^a^	0.24	[0.148, 0.331]
**COI**_**HE**_				
**Cortical thickness**				
Frontal pole LH	7.67	0.006**^a^	0.122	[0.032, 0.212]
Fusiform RH	15.43	0.000***^a^	0.195	[0.098, 0.292]
Inferior temporal RH	8.29	0.004**^a^	0.187	[0.084, 0.289]
Isthmus cingulate LH	10.39	0.001***^a^	0.214	[0.115, 0.313]
Isthmus cingulate RH	8.52	0.004**^a^	0.184	[0.087, 0.281]
Lingual RH	9.74	0.002**^a^	0.213	[0.115, 0.311]
Medial orbitofrontal RH	19.82	0.000***^a^	0.236	[0.146, 0.327]
Paracentral RH	10.9	0.001***^a^	0.195	[0.104, 0.285]
Pars triangularis RH	13.73	0.000***^a^	0.223	[0.126, 0.321]
Posterior cingulate RH	17.93	0.000***^a^	0.268	[0.172, 0.364]
Precuneus LH	11.9	0.001***^a^	0.224	[0.13, 0.318]
Precuneus RH	11.04	0.001***^a^	0.199	[0.105, 0.293]
Rostral anterior cingulate RH	11.71	0.001***^a^	0.232	[0.14, 0.324]
Rostral middle frontal LH	10.96	0.001***^a^	0.164	[0.066, 0.263]
Rostral middle frontal RH	28.14	0.000***^a^	0.307	[0.213, 0.4]
Superior frontal LH	16.99	0.000***^a^	0.176	[0.079, 0.274]
Superior frontal RH	25.64	0.000***^a^	0.258	[0.164, 0.352]
Temporal pole LH	11.73	0.001***^a^	0.192	[0.103, 0.28]
**Subcortical volume**				
Pallidum LH	13.09	0.000***^a^	−0.188	[−0.281, −0.094]
Ventral DC LH	9.76	0.002**^a^	−0.15	[−0.252, −0.049]
**COI**_**SE**_				
**Cortical thickness**				
Medial orbitofrontal RH	8.18	0.004**	0.199	[0.103, 0.294]
Rostral middle frontal RH	14.56	0.000***^a^	0.282	[0.187, 0.378]
**Subcortical volume**				
Caudate LH	8.08	0.004**^a^	−0.079	[−0.181, 0.024]
Pallidum LH	15.73	0.000***^a^	−0.198	[−0.295, −0.1]
Ventral DC LH	12.15	0.000***^a^	−0.134	[−0.238, −0.031]

Regions of interest (ROIs) were corrected separately for each modality (e.g., cortical thickness, subcortical volume) and accounted for the number of tests conducted (e.g., two-way and three-way interactions). Total corrections for cortical ROIs = 136 Total corrections for subcortical ROIs = 32. LH, left hemisphere; RH, right hemisphere; COI, child opportunity index; HE, health/environmental, ED, education; SE, social/economics. ^***^p <0.001; ^**^p <0.01, ^a^Benjamini-Hochberg FDR corrected.

#### COI_HE_

Independent of time, BMI was significantly moderated by COI_HE_ (e.g., access to resources such as healthy food stores, green spaces, walkability; Figure 4B) in 18 cortical and 2 subcortical brain regions. COI_HE_ showed the largest moderating effects on BMI with cortical thickness in the right rostral middle frontal [*F*_(1, 2, 513.4)_ = 28.2, *p* < 0.001], superior frontal [*F*_(1, 2, 749.7)_ = 25.6, *p* < 0.001], medial orbitofrontal [*F*_(1, 1, 892.3)_ = 19.8, *p* < 0.001], and posterior cingulate [*F*_(1, 2, 460.1)_ = 17.9, *p* < 0.001], and the left superior frontal gyrus [*F*_(1, 2, 789.5)_ = 17.0, *p* < 0.001]. A list of all brain regions (and the betas from the interaction term) that were moderated by COI_HE_ are presented in Table 4. The overall pattern suggested that youth with access to fewer health/environmental (i.e., lower COI_HE_) opportunities had greater BMIs, thinner cortices across time points, and larger subcortical volumes. Visual representations for the interactions for the two largest moderation effects (e.g., rostral middle frontal, superior frontal) are presented in Figure 3B. These moderation effects (e.g., ${\text{COI}}_{\text{HE}}^{*}$Brain on BMI) were independent of Youth sex, Youth race, Youth ethnicity, puberty, income-to-needs, time (e.g., baseline, 2-year follow-up), and the child's access to COI_ED_ and COI_SE_ resources in the neighborhood.

#### COI_SE_

Regardless of time, a child's access to social/economic (COI_SE_) resources in the neighborhood moderated the strength of the relationship between BMI and cortical thickness in the right rostral middle frontal gyrus [*F*_(1, 3, 079.3)_ = 14.6, *p* < 0.001] and subcortical volume of the left caudate [*F*_(1, 4, 705.2)_ = 8.1, *p* = 0.004], pallidum [*F*_(1, 3, 353.7)_ = 15.7, *p* < 0.001], and ventral DC [*F*_(1, 3, 700.9)_ = 12.2, *p* < 0.001, Figure 4C]. Cortically, youth with lower COI_SE_ resources showed a steeper negative relationship between greater BMI and thinner cortices, when compared to youth with medium and higher levels of COI_SE_. Subcortically, youth with access to fewer COI_SE_ resources had larger left hemisphere subcortical volumes of the caudate, pallidum, and ventral DC. Figure 5C shows visual representations of these interactions for the two largest effects of which were 1 cortical (e.g., right rostral middle frontal) and 1 subcortical region (right pallidum). These effects were independent of Youth sex, Youth race, Youth ethnicity, puberty, income-to-needs, time (e.g., baseline, 2-year follow-up), and the child's access to COI_HE_ and COI_ED_ resources in the neighborhood.

### Brain regions with non-overlapping interactions

There were some brain regions that showed non-overlapping two-way interactions with each of the COI domains (Figure 4D). In other words, these brain regions only showed significant associations with one specific COI domain and not the other two. The association between BMI and right temporal pole cortical thickness was significantly moderated by COI_ED_ but not COI_HE_ nor COI_SE_. Brain regions with significant two-way interactions unique to COI_HE_ consisted of the right superior frontal, fusiform, pars triangularis, rostral anterior cingulate and middle frontal, paracentral, lingual, inferior temporal gyri, and left precuneus, rostral middle frontal, isthmus cingulate, and frontal pole gyri. The subcortical volume of the left caudate was the only region that was significantly moderated by COI_SE_ but not COI_HE_ nor COI_ED_.

## Discussion

Although single attributes of SEDs are related to obesity risk and brain development (4, 6, 7, 10, 11, 27, 50), no studies have considered how multidimensional attributes of the environment may explain these relationships, especially within a longitudinal framework. The current study highlights the influence of 3 child-specific environmental resources (e.g., education, healthy/environmental, economics) (18) on the relationship between BMI and brain development during a 2-year period in adolescence. Our results demonstrate that social/economic and educational resources in the community may contribute to unhealthy weight gain trajectories independently from financial resources within the family (e.g., income-to-needs), and that these weight changes may, in turn, marginally affect brain structure over a 2-year period in adolescence. COI_HE_ largely moderated the associations between the brain and BMI, but these relationships were relatively stable, meaning that there were no changes over time. This suggests that access to health/environmental resources in the community play a large role in explaining the relationship between the brain and BMI, but, in the age range of youth studied here, they do not continue to contribute to unhealthy weight gain trajectories and greater brain alterations during pre/early adolescence. Importantly, given the number of brain regions associated with COI_HE_ relative to the other two, these specific environmental resources may have a larger influence on BMI and brain relationships than access to quality COI_SE_ and COI_ED_ resources. This finding is important from a public policy standpoint because this suggests that access to health promoting resources and less environmental toxicity (components measured by COI_HE_) may be important areas to target to decrease obesity rates in deprived neighborhoods. All findings were independent of the amount of money available to the family (e.g., income-to-needs, Figure 1E), which further highlights the importance of the opportunities available in the neighborhood environment for child health outcomes, like obesity.

For several reasons, the analyses focused on how SED moderate the relationship between body mass index (BMI) and structural brain development during adolescence. First, altered brain structure has been observed in youth with obesity (24), suggesting that one reason for overeating is impaired neural signaling around food choice. Because the brain plays a key role in food-intake regulation (25), it is imperative to understand more about the brain-environment relationships that may continue to escalate obesity risks. Second, SED also impact brain development, and inadequate resources have been correlated with altered brain structure as well (7, 26, 27). However, it is not known how each of these domains may further interact with the brain to influence obesity risk. Moreover, to our knowledge, no studies have looked at how multiple environmental factors relate to obesity risk and brain structure development over time. Therefore, it is not known whether these associations are stable (e.g., social and environmental aspects have a general effect that does not worsen over time) or if they have a sustained impact throughout development. To address these questions, we utilized data from the baseline and 2-year follow-up from the Adolescent Brain Cognitive Development study (ABCD Study^®^), a 10-year longitudinal neuroimaging study in 11,878 American youth (28).

Independent of income within the family, youth with lower COI_SE_ (lower socioeconomic resources) were even heavier at the 2-year follow-up then they were at baseline, and this increase in BMI corresponded to greater subcortical volume of the caudate nucleus bilaterally. The COI_SE_ focuses on assessing amenities in the neighborhood, like access to parks, after school recreational activities and more medical and community service providers (18), which may have protective affects against obesity maintenance and acceleration. Thus, a lack of access to these resources could have implications for weight gain, which could be one reason to explain the longitudinal associations between unhealthy weight gain trajectories and altered caudate structure. The caudate has been implicated in how the brain responds to food rewards, obesity, and weight gain in youth and adults (51–53), suggesting that heightened reward responses may cause individuals with obesity to overeat. In our study, it may be that a lack of social/economic resources in the environment facilitate continued weight gain, and that weight gain itself caused structural changes in the brain in the caudate (a reward region) that facilitate overeating. This is in line with other studies using the ABCD Study^®^ data showing that neighborhood resources are related to weight gain (23) and, that weight gain is associated with changes in brain structure (54, 55). Others postulate that social/economic resource advocacy may play a role in determining how much funding is available for neighborhoods (56), while other studies suggests that decreased social/economic neighborhood resources are detrimental to healthy development of children and adolescents (57), that have long-lasting (and even lifetime) effects (58). Thus, providing additional social/economic resources to disadvantaged communities may help to negate continuous weight gain during adolescence. However, future studies are needed to examine how these resources may lessen the impacts of weight gain over time.

Importantly, our finding that the higher BMI corresponded with increased caudate volume is in contrast to studies showing that caudate volume decreases with pubertal acceleration (59) and aging (60–62). Caudate volume increases during childhood (62), but then declines during the later stages of puberty (e.g., Tanner staging 4–5) (63), which occurs later for males (62, 63). Thus, in our study, increases in volume of the caudate could be due to acceleration of pubertal development as youth with obesity who undergo pubertal changes at an earlier age than those of a healthy weight (64), but the directionality of these effects remain controversial (e.g., does obesity cause early puberty, or does early puberty increase obesity). However, it could be that obesity disrupts normal developmental trajectories as studies have shown that youth and adults with obesity have larger subcortical volumes (24, 65) and greater impulsivity (66) which may drive greater food intake. Other studies have noticed increases in caudate volume following electroconvulsive treatment of depression of adults with normal weight and obesity (67) and in treatment studies with anorexia (68). Yet, the mechanisms driving increased caudate volume are not clear, as not all studies show an association with obesity and increased caudate volume. To our knowledge, besides our study, no studies have examined how unhealthy weight gain trajectories alter brain structure.

Lower (greater deprivation, fewer resources) COI_SE_ and COI_SE_ were also related to greater BMs at the 2-year follow-up. Youth with lower COI_SE_ and COI_SE_ had accelerated decreases in cortical thinning of the bilateral occipital cortex when compared to baseline and to those youth who had greater COI_SE_ and COI_SE_. Altered structural and functional responses in the lateral occipital cortex have been observed in youth with obesity (69–71), suggesting that obesity corresponds with altered visual attentional bias to food. The occipital cortex is not commonly associated with socioeconomic status or other measures of resource deprivation, like the area deprivation index [i.e., a multidimensional assessment of poverty (4)], so what this means of functioning over time warrants further investigation. However, it is hard for us to compare our results with cross-sectional studies that used other types of resource assessment (4, 22). Thus, our findings highlight the importance of the community's social/economic and access to quality education resources to prevent obesity rates from increasing over time.

Overall, the moderating effects of COI_ED_ and COI_SE_ on the longitudinal association between brain structure and BMI were minimal. This was surprising as the literature highlights how important within-family income and education and neighborhood poverty and resources (e.g., poverty rates) (4, 10), are for predicting childhood obesity. While we were expecting to see larger associations of these educational and economic resources on longitudinal associations between the brain and BMI, there could be at least two explanations for this. First, the COI assesses neighborhood economic resources that are specifically pertinent to the child (such as money devoted to parks, extracurricular activities) (18), whereas other studies have examined just the impacts of community poverty, within-family economic resources, or parent education (4, 10, 11, 22, 72). Second, when assessing the moderating effects of social/economic resources, we controlled for other environmental resources (e.g., COI_HE_, COI_ED_) and income factors (e.g., income-to-needs), so that we could isolate the unique variance associated with socioeconomic factors while also accounting for any other resources that may counteract any potential detrimental effects of socioeconomics resources on BMI. Therefore, our findings suggest that when compared amongst these other factors, neighborhood economic and social resources may carry little weight, especially when understanding how the neighborhood impacts childhood obesity development over time. From a public policy perspective, this is an encouraging finding because it allows policy to focus on supporting resource efforts for other domains that may have a greater impact, like increasing support for health/environmental resources. Additionally, this finding can help to assuage the stigma associated with socioeconomics and childhood obesity; as in our study, socioeconomics (neighborhood or family-level) was not the largest factor driving the BMI and brain relationships over time.

Given that we did not observe any changes between the COI_HE_ and cortical thickness or subcortical volume over time, it is not likely that access to health/environmental resources (or lack thereof) exacerbate brain/BMI relationships. However, there were widespread effects of this domain on the relationship between the brain and BMI at both time points suggesting some relative stability of these relationships, at least at this point in development. Youth with greater COI_HE_ deprivation were heavier, and this corresponded to thinner cortices in several frontal regions (e.g., bilateral rostral middle frontal, superior frontal, cingulate) as well as larger subcortical volumes (e.g., left pallidum and ventral DC). This finding is in line with the general body of literature showing a relationship between the physical environment and child obesity development (73, 74). From this perspective, studies have evaluated the effects of traffic noise, air pollution, walkability, and accessibility and availability of parks and playgrounds, but, again, all these studies have largely treated each of these factors in isolation. It is important to note that the health/environmental domain also included assessments of the natural environment, which includes measurements of air pollution and other toxicants. Environmental toxicants have been linked to decreases in cognitive functioning (75) and brain structure alteration (76), so understanding how it relates to obesity and brain structure are equally important. At this point, we cannot confer whether access to grocery stores vs. air pollution had a bigger impact on the relationship between the brain and BMI, so future studies are needed to disentangle these factors. However, what our results do suggest is that neighborhood infrastructure for health promoting development, which encompasses environmental toxicants, has a robust effect on the relationship between the brain and BMI from ages 9–12 years.

There were also regions that showed unique associations with each domain. For example, a positive association between the left caudate volume and BMI was only moderated by COI_SE_, whereas only COI_ED_ moderated the relationship between cortical thinning in the right temporal pole and increased BMI. Naturally, due to the extent of our findings with COI_HE_, there were several regions that were uniquely moderated by this domain. Notably, many of these regions have been implicated in food intake reward processing and cognitive control (e.g., pars triangularis, prefrontal cortex, cingulate) (77). Future studies are needed to understand the implications of these findings.

### Strengths and limitations

There were several strengths of this study. This was the first study to evaluate the longitudinal associations between several facets of environmental resources on the relationships between the brain and BMI. This study also established the unique effects of the environmental resources on brain and BMI relationships independent of other potential beneficial environmental factors. However, there were also several limitations that serve as future directions. First, the ABCD Study^®^ did not collect assessments of actual food intake, so it is hard to infer what our results mean regarding actual overeating mechanisms. Second, COI_HE_ encompassed physical structure and natural environmental attributes, so we are limited in the conclusions that we can make when interpreting the magnitude of effects that each of these sub-attributes have on the brain and BMI relationships. Lastly, this paper included caregiver reported race and ethnicity as covariates in the model, which may be removing some variance explained by the COI, due to environmental injustices and systemic racism that has caused minoritized populations to live in more disadvantaged neighborhoods (14). Here, we chose to include caregiver reported race and ethnicity to adjust for social inequalities that may have disproportionately affected some populations, as this marginalization has been associated with both greater resource deprivation and increased BMI. Given research suggesting that there are differences in body composition by various caregiver reported racial and ethnic groups (47), past research has included race (or ethnicity) as a confounding variable in analysis (48). However, race and ethnicity are social constructs (78) that cannot provide insight into historical racism, culture nor body fat composition in an accurate fashion. Because the ABCD Study^®^ did not collect more objective measures, we were limited in our ability to capture the variance associated with these metrics (e.g., racism, culture, body fat) without including the socially constructed variable of race and ethnicity. We note the limitations of inclusion of these variables and advocate for future studies to collect more accurate and objective measurements to account for the variability of experience and body composition (e.g., DEXA, bioelectrical impedance) across different groups of people to provide a better picture of metabolic risk that is not defined by social constructs.

## Conclusion

Our results especially highlight the importance of the child's access to health/environmental resources within their communities, perhaps more so than education and social/economic resources, independent of any of the financial resources that are directly available to the family (i.e., income-to-needs). This is important because previous research has suggested that family income is an important determinant of obesity during childhood. Although family income may be important, here we showcase the substantial effects of neighborhood resource access on brain development and increasing weight gain trajectories. Collectively our findings demonstrate that neighborhood SEDs for health-promoting resources play a particularly important role in moderating relationships between brain and BMI in early adolescence regardless of family-level financial resources. These findings have implications for public policy makers who may wish to tailor their efforts to increasing health/environmental resources as a potential prevention for obesity development during childhood.

## Data availability statement

The publicly available dataset can be found at: These data can be found at: nda.nih.gov/abcd.

## Ethics statement

The study involving human participants were reviewed and approved by UC San Diego. Written informed consent to participate in this study was provided by the participants' legal guardian/next of kin.

## Author contributions

SA wrote the manuscript and performed the analyses with the guidance of ES and AM. SA conceptualized and curated the data with the help of ES. ES provided funding for the project as part of the ABCD Study^®^ consortium. EK and AM contributed to interpretation and data analysis. The ABCD Study^®^ consortium investigators designed and implemented the study and/or provided data but did not participate in the analyses or writing of this manuscript. All authors contributed feedback, read, and approved the final manuscript.
