# Supplementary material for: Access to quality health resources and environmental toxins affect the relationship between brain structure and BMI in a sample of pre and early adolescents
Source: Front Public Health. 2022 Dec 15;10:1061049. doi: 10.3389/fpubh.2022.1061049 (PMC9797683; doi:10.3389/fpubh.2022.1061049)
Supplement: Supplementary file 1 [file Data_Sheet_1.PDF]

Table S1. The number of subjects available based on each exclusion criterion applied.

	**n**
Y2 available BMI data	
Overlap with BMI data at Y2	7697
No BMI measurement errors or outliers	7166
Not underweight	6763
No eating disorders	5590
Complete covariate data	4032
Valid residential address	3886
Passed T1 QC at Y2	3353
Passed above criteria also at baseline	3248
No siblings	2914
Available data for COI	2750
Final sample	

Note. Metrics were generated starting with a dataframe that consisted of baseline demographics and year 2 neuroimaging data. After quality control assessments of the year 2 data, the dataframe was merged with all available demographic and neuroimaging data from the baseline assessment (see line "Available data for all metrics …"). Y2= years 2; BMI = Body mass index.

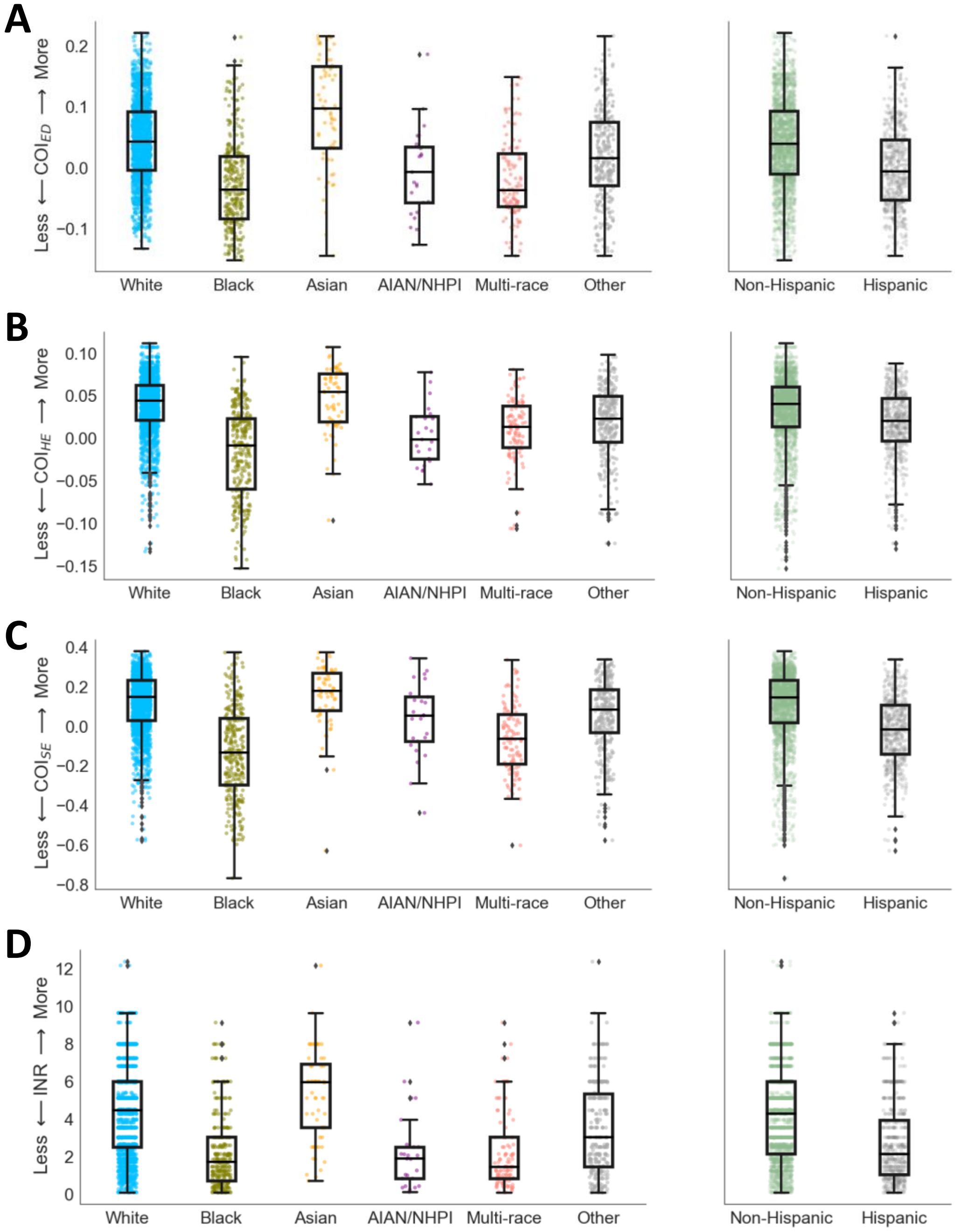

Figure S1. Distributions of resources for each neighborhood-level domain (**A, B, C)** and family-level income-to-needs (**D**) across race and ethnicities. COI = Child Opportunity Index. ED = education domain; HE = health/environmental domain; SE = social/economics domain; INR = income-to-needs ratio; AIAN/NHPI = American Indian Alaska Native / Native Hawaiian Pacific Islander. Other corresponds to families that did not identify as any of the other 5 provided racial categories.
